# RNA-guided CRISPR-Cas technologies for genome-scale investigation of disease processes

**DOI:** 10.1186/s13045-015-0127-3

**Published:** 2015-04-02

**Authors:** Sean E Humphrey, Andrea L Kasinski

**Affiliations:** Department of Biological Sciences, Purdue University, 1203 West State Street, West Lafayette, IN 47907 USA

## Abstract

From its discovery as an adaptive bacterial and archaea immune system, the clustered regularly interspaced short palindromic repeats (CRISPR)-Cas system has quickly been developed into a powerful and groundbreaking programmable nuclease technology for the global and precise editing of the genome in cells. This system allows for comprehensive unbiased functional studies and is already advancing the field by revealing genes that have previously unknown roles in disease processes. In this review, we examine and compare recently developed CRISPR-Cas platforms for global genome editing and examine the advancements these platforms have made in guide RNA design, guide RNA/Cas9 interaction, on-target specificity, and target sequence selection. We also explore some of the exciting therapeutic potentials of the CRISPR-Cas technology as well as some of the innovative new uses of this technology beyond genome editing.

## Introduction

**Figure 1:**
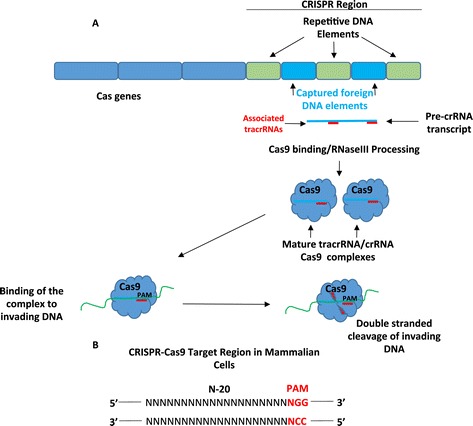
**The CRISPR-Cas9 bacterial immune system and design of a CRISPR-Cas9 target sequence. (A)** The CRISPR-Cas system acts as an adaptive immune system in bacteria and archaea. Clustered regularly interspaced short palindromic repeats (CRISPR) regions are stretches of repetitive genomic bacterial or archaea DNA interspersed by segments of foreign DNA sequences captured from bacterial phages and plasmids. A cluster of Cas (CRISPR associated) genes are located near the CRISPR region. The Cas9 gene, which is unique to type II CRISPR systems, codes for an RNA-guided endonuclease. Following foreign DNA infection in type II CRISPR systems, the CRISPR region is transcribed as a single RNA transcript called a pre-crRNA, and in type II systems, the pre-crRNAs are bound by tracrRNAs, processed into individual crRNA:tracrRNA duplexes by RNase III and form a complex with Cas9. The crRNA sequences are complementary to the foreign DNA and direct the Cas9 nuclease to form a complex with the foreign DNA and introduce a double-stranded break. **(B)** CRISPR-Cas9 target sequences are 20-nt long and are flanked by a protospacer adjacent motif (PAM) sequence in the form of 5′-NGG.

Clustered regularly interspaced short palindromic repeats (CRISPR)-Cas systems are adaptive immune systems used by many bacteria and archaea to fight off foreign DNA in the form of bacterial phages and/or plasmids [1-5]. Although multiple CRISPR-Cas types (I, II, and III) and subtypes (I-A to I-F) have been identified in bacteria and archaea, we pay particular attention to the specifics of the type II since type II has been engineered and adapted for use in eukaryotic systems, which is the focus of this review. Generally, the CRISPR-Cas system works through RNA-directed endonuclease cleavage of the invading genomic sequence. The invading sequence is captured and inserted directly into the genome of the host organism between CRISPR regions (Figure 1A) [6-8]. Following foreign DNA infection, the sequences within the CRISPR regions are transcribed as a single RNA transcript called a precursor CRISPR RNA (pre-crRNA). In the CRISPR-Cas9 system, the pre-crRNAs are bound by additional RNAs termed transactivating CRISPR RNAs (tracrRNAs) [9-12]. Once bound, the pre-crRNAs are processed into individual crRNA:tracrRNA duplexes by RNase III and together form a complex with an endonuclease [9-12]. The endonuclease Cas9 that is encoded from a region of the host genome adjacent to the CRISPR region is directed to the invading DNA in a sequence-dependent manner via the crRNA. Once bound to the foreign DNA, Cas9 introduces a double-stranded break in the foreign DNA [11-13].

Realizing the potential power of a programmable nuclease to edit mammalian genomes, the CRISPR-Cas9 system has since been commercially developed as a technology for use in loss-of-function (LOF) studies [13,14]. Regardless of the platform, a tracrRNA, a mammalian optimized Cas9 protein, and a small guide RNA (sgRNA) that is analogous to the crRNA must be expressed at minimum. In some engineered systems, the sgRNA and the tracrRNA are expressed separately while in others, they are expressed as a single transcript [14-16]. The sgRNA sequences are generally 20-bp long, but sgRNA sequences of 17–18 bp are also effective [17]. Target sequences must be adjacent to a protospacer adjacent motif (PAM) sequence in the form of 5′-NGG (Figure 1B) [18-20]. Once expressed in cells, the Cas9 protein, tracrRNA, and the sgRNA form a complex, bind to the target sequence, and make a double-stranded break in the target. The break is repaired via the cellular process of nonhomologous end joining (NHEJ), an error-prone process that introduces insertion, deletion, and frameshift mutations into the target sequence. Targeted mutations can also be introduced by cotransfecting single- or double-stranded oligodeoxynucleotides to promote homology-driven repair [21-28].

In studying the molecular signaling pathways that impinge on disease processes, many large- and small-scale expression studies of diseased tissues have provided extensive lists of genes that are aberrantly expressed in diseases such as cancer [29]. Such studies have greatly advanced our knowledge about the gene expression signatures of disease and provided us with a wealth of genes that are important predictive and prognostic biomarkers. The challenge moving forward is how to effectively separate the genes that are ‘drivers’ of disease from gene ‘passengers’ whose aberrant expression has no relevance to the disease state. Loss-of-function studies are an effective way to assess whether a gene is a driver of disease or a passenger.

The CRISPR-Cas system has some important advantages over other methods in LOF studies. At present, a widely used method for knocking down the expression of genes is through the induction of RNA interference (RNAi) either through transfection or viral transduction of small interfering RNAs (siRNA) or short hairpin RNAs (shRNA) [30-34]. While the use of RNAi has certainly advanced many fields, there are also some inherent drawbacks. First, siRNAs and shRNAs must be continuously expressed for longitudinal studies, which can lead to confounding off-target effects and false negatives [35]. Second, knockdown by these methods can often be incomplete [36,37]. Gene knockout technologies, such as those mediated by CRISPR-Cas9, overcome such deficits by knocking out individual gene expression at the genomic level and do so with minimal off-target effects. And while RNAi is limited to inhibiting the function of RNAs, CRISPR-Cas9 technology can be used to introduce random or targeted mutations into any portion of the genome such as the coding region, promoter, or enhancer regions of genes [38].

Importantly, the CRISPR-Cas9 system has recently been developed into a tool for genome-scale loss-of-function screens by several laboratories. In this review, we examine and compare these recently developed genome-scale technologies. We also explore some of the potential therapeutic and innovative new uses of the CRISPR-Cas technology beyond genome editing.

### Genome-scale CRISPR-Cas9 platforms (single Cas systems)

The various genome-scale CRISPR-Cas9 platforms target genes by directing either a single Cas9 protein to each targeted gene (referred to here as a single Cas system) or by directing two Cas9 proteins to each targeted gene (referred to here as a dual Cas system). Shalem and colleagues recently developed a single Cas lentiviral genome-scale CRISPR-Cas9 KnockOut (GeCKO) library [39]. Each lentiviral vector in the library delivers stable expression of Cas9, a specific sgRNA generated against a single gene, and a puromycin selection marker to the cell. In total, 64,751 unique lentiviral vectors make up the GeCKO library that target 18,080 human genes. This powerful new method of interrogating gene function on a genome-wide scale was used in both positive and negative selection screens in human cells. First, to test the ability of the GeCKO library to identify genes critical to survival, a negative selection screen was conducted by transducing a human melanoma cell line and a human stem cell line with the GeCKO library. Comparing the deep sequenced population of sgRNAs present in the initially transduced cells to the deep sequenced population of sgRNAs present in cells that survived 14 days post transduction revealed a reduction in sgRNA diversity due to the depletion of sgRNAs targeting genes critical for survival. Importantly, this type of negative selection screen not only allows for the identification of gene sets that are critical to the survival of both cancer and noncancer cells but also identifies gene sets that are unique to the survival of cancer cells and thus genes that are possible therapeutic targets. GeCKO was also effective in a positive selection screen that enriched for sgRNAs that knock out genes essential for chemotherapeutic response. A375 melanoma cells, which are sensitive to the B-RAF serine/threonine protein kinase inhibitor vemurafenib, were transduced with the GeCKO library and grown in the presence of vemurafenib. After 14 days, a group of cells had been rendered drug resistant. Deep sequencing of the population of sgRNAs present in the drug-resistant cells versus the vehicle-treated cells revealed the enrichment of multiple sgRNAs directed against a subset of genes, suggesting that loss of those particular genes likely contribute to vemurafenib resistance. Importantly, this type of unbiased loss-of-function assay identified several genes not previously implicated in vemurafenib resistance, which opens up completely new avenues of research into the mechanisms of vemurafenib resistance. Recently, vast improvements have been made to the GeCKO library platform [40]. First, a newly modified vector, lentiCRISPRv2, was generated that displays a tenfold increase in viral titer over the original lentiCRISPRv1 vector. The modified vector, lacking one of two nuclear localization signals contained in the lentiCRISPRv1 vector, has been human-codon optimized, and the U6-driven sgRNA cassette has been repositioned. Secondly, to increase viral titer even further, a two-vector system was generated in which Cas9 and sgRNAs are expressed from separate viral vectors (lentiCas9-Blast and lentiGuide-Puro) with distinct antibiotic selection markers. LentiGuide-Puro has a 100-fold increase in viral titer over the original lentiCRISPRv1 vector. Also, the number of human genes targeted by the sgRNA library was increased to 19,050, guide RNAs targeting 1,864 miRNAs were included, and a sgRNA library against 20,611 mouse protein-coding genes and 1,175 miRNAs was also generated. Importantly, these powerful new reagents are available to the academic community through Addgene.

A similar large-scale study also utilized the CRISPR-Cas9 system to develop a lentiviral library containing 73,000 sgRNAs against 7,114 genes that was used for genetic screens in human cells [41]. The library was transduced into cells stably expressing Cas9. This library proved effective in positive and negative selection screens at identifying genes important to the mismatch repair pathway and genes required for cellular proliferation. Critical parameters for designing effective sgRNAs were also identified in this study. Careful dissection and evaluation of the sgRNAs revealed that guide sequences with very high or low GC content were less effective against their targets, sgRNAs targeting the last coding exon were less effective than those targeting earlier exons, and sgRNAs targeting the sense strand were less effective than those targeting the antisense strand. The authors further hypothesized that differences in sgRNA efficacy might also result from sequence features governing the interaction between the sgRNA and Cas9. To test this, a method to profile the sgRNAs directly bound to Cas9 in a highly parallel manner was developed. By comparing the abundance of sgRNAs bound to Cas9 relative to the abundance of corresponding genomic integrants, it was found that the nucleotide composition near the 3′ end of the spacer sequence was the most important determinant of Cas9 loading. Cas9 preferentially bound sgRNAs containing purines in the last four nucleotides of the spacer sequence over sgRNAs containing pyrimidines in the same location. Using this algorithm, a whole-genome sgRNA library was developed with great promise for use in future genome-scale screening.

An additional genome-wide lentiviral CRISPR-sgRNA library was generated containing 87,897 sgRNA targeting 19,150 mouse genes for comprehensive loss-of-function screening in mice [42]. A lentiviral vector was used to express the sgRNAs in embryonic stem cells stably expressing Cas9. A functional screen using this platform revealed 27 known and 4 previously unknown genes that modulate toxin susceptibility demonstrating the power and utility of CRISPR-Cas-based genome-wide loss-of-function screening.

One concern of the single Cas systems is the potential of off-target mutations introduced by the binding of CRISPR-Cas9 to target sequences that may appear more than once in the genome or to sites similar enough to the target sequence that allow binding of the sgRNA [43-45]. To examine the degree of off-targeting in the GeCKO library, developed by Shalem and colleagues [39], on- and off-target allele modification frequencies were measured by deep sequencing the targeted region at 3–5 predicted potential off-target genomic regions for each of 12 different sgRNAs. Near 100% allele modification in the targeted regions for all 12 sgRNAs was verified with only 3 sgRNAs showing appreciable modification to one or more of the predicted off-target regions (Table 1). Wang and colleagues also analyzed off-target activity in their lentiviral CRISPR-Cas library [41]. Cas9 and a guide RNA directed at the adeno-associated virus integration site 1 (AAVS1) locus were stably expressed in a near-haploid human chronic myelogenous leukemic cell line cells for 2 weeks. The sgRNA target region along with 13 predicted potential off-target sites were examined by high-throughput sequencing. Cleavage of the target region occurred 97% of the time with off-target sites mutated 0–2.5% (Table 1). The authors note that the one off-target site with appreciable cleavage had perfect complementarity to the terminal 8 bp of the sgRNA and that on average, all sgRNAs are predicted to have 2.2 such sites in the genome that almost always occur in noncoding areas of the genome. An additional study tested two sgRNAs for each of 26 genes [42]. Deep sequencing revealed that 50 of the 52 sgRNAs analyzed were able to induce double-stranded breaks in the target genes with variable cutting frequencies (Table 1) with relatively low off-targeting (Table 1).

**Table 1:** **Summary of reported on- and off-target mutation frequencies of single and dual CRISPR-Cas9 systems**

**Investigators**	**Platform**	**Reported on-targeting (%)**	**Reported off-targeting (%)**
Shalem et al., 2014 [39]	Single Cas platform	~100	0–15^b^
Wang et al., 2014 [41]	Single Cas platform	97	0–29.5^c^
Koike-Yusa et al., 2014 [42]	Single Cas platform	12.7	~0.7^d^
Tsai et al., 2014 [46]	Dual Cas platform	3–40	0
Ran et al., 2013 [48]	Dual Cas platform	40^a^	0
Guilinger et al., 2014 [47]	Dual Cas platform	8–22	0

^a^On-target mutation frequencies of up to 40% were observed in target sequences when the distance between the Cas9 nickase pairs were between −4 and +20 bp.

^b^9 of 12 sgRNAs showed minimal (~0–3%) modification of predicted off-target sites. 3 of 12 sgRNAs showed modification (~15% or higher) on at least 1 of their off-target sites.

^c^0–2.5% cleavage of 12 of the 13 predicted off-target sites, 29.5% cleavage of 1 of the 13 predicted off-target sites.

^d^Cleavage analysis of 275 potential off-target sites for the *Pigga* site 2 sgRNA revealed only 2 of those potential off-target sites were cleaved and both were in noncoding regions (2/275*100 = ~0.7%).

### Genome-scale CRISPR-Cas9 platforms (dual Cas systems)

**Figure 2:**
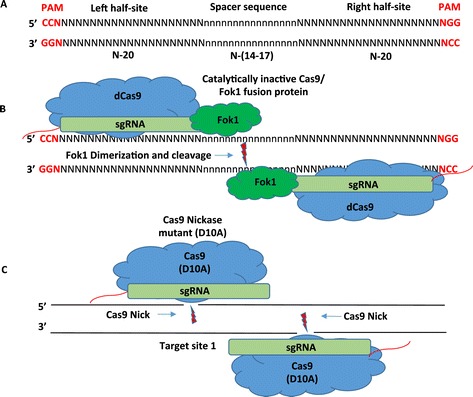
**Dual CRISPR-Cas technologies that increase nuclease specificity. (A, B)** Dimeric CRISPR RNA-guided Fok1 nuclease target sequences consist of two 20 nt half-sites flanked by a protospacer adjacent motif (PAM) sequence in the form of 5′-NGG that are separated by a 14–17 nt spacer sequence. Each half-site is bound by a Cas-9/Fok1 fusion protein. Once bound, the Fok1 domains of two different Cas-9/Fok1 fusion proteins dimerize and introduce a double-stranded break in the spacer sequence. **(C)** Dual RNA-guided CRISPR-Cas9 nickase system. In this system, two sgRNAs are expressed that each guide a mutant version of Cas9 (Cas9-D10A) (that only nicks one strand of the DNA rather than making a double-stranded cut) to two different sequences that flank the target region. The two Cas9 nickases bind to opposite strands of the DNA nicking both DNA strands flanking the target region. This introduces a site-specific double-stranded break that is then repaired by NHEJ.

The low but still measurable frequency of off-target mutations in the single Cas systems discussed above has been improved to essentially undetectable levels by the development of dual cas platforms (Table 1). To increase the specificity of RNA-guided nucleases, the dimerization-dependent Fok1 nuclease domain was fused to a catalytically inactive Cas9 (dCas9) protein [46]. In this system, sequence-specific DNA cleavage only occurs upon dimerization of two Fok1 nuclease domains from two different RNA-guided Fok1 nucleases (RFNs) that are bound in close proximity to two unique target sites (called half-sites) (Figure 2A, B). To be fully effective, the half-sites must be 14–17 bp apart and the entire target sequence must be flanked on the 5′ end by the sequence 5′-CCN and flanked on the 3′ end by the sequence NGG-3′. This system requires the use of two sgRNAs (one for each half-site). As the authors note, a full 44 bp RFN target site would almost always be unique in the genome unless located in a duplicated area of the genome. However, to assess the potential of off-targeting by RFNs, all sites in the genome that most closely matched the target regions of 3 RFNs were identified. Deep sequencing analysis of these areas following RFN-directed target mutagenesis detected no mutations above background (Table 1). This data suggests that RFN technology offers extraordinary precision. On-target mutation frequencies induced by RFNs at 12 different target sites in 9 different human genes ranged between 3 and 40% (Table 1). Some important cost advantages to this platform are that the plasmids to express the Cas9/Fok1 fusion proteins and the sgRNAs are inexpensive to purchase and the software to locate suitable target sequences against a gene of interest is publicly available. However, if one is generating an extensive library of RFNs against hundreds or thousands of genes, then the additional costs of generating two sgRNAs per target for the RFN technology may be a concern [46].

A separate dimeric CRISPR RNA-guided Fok1 nuclease architecture was also recently developed [47]. Four configurations of the Fok1 nuclease, dCas9, and nuclear localization signal (NLS) were generated and tested for DNA cleavage. Of the four, only the NLS-Fok1-dCAS9 architecture generated a high frequency of cleavage. And although the NLS-Fok1-dCas9 (fCas9) system was shown to modify target sequences with lower efficiency than wild-type Cas9, the ratio of on-targeting/off-targeting was higher than that of wild-type Cas9 and paired Cas9 nickases proving it to be a technology with a very high degree of specificity (Table 1). fCas9 was shown to effectively cleave target sites in which the sgRNA binding sites were spaced 15–25 bp apart giving a more flexible range than the 14–17 bp spacing requirement of the RFNs. Though this system has not yet been developed into a genome-scale editing system, the authors report that target sites conforming to the substrate requirements of fCas9 occur on average every 34 bp suggesting it has the versatility to be developed into a genome-wide editing approach.

Another strategy employed to address the issue of CRISPR-Cas9 specificity makes use of a mutant version of Cas9 (Cas9-D10A) that only nicks one strand of the DNA rather than making a double-stranded cut [48]. In this system, two sgRNAs are expressed that each guides a Cas9-D10A nickase to two different sequences that flank the target region (Figure 2C). The two Cas9 nickases bind to opposite strands of the DNA. Nicking of both DNA strands introduces a site-specific double-stranded break that is then repaired by NHEJ. Similar to the RFN technology, the use of two sgRNAs instead of one to introduce a double-stranded break has the potential to greatly increase specificity. However, a potential caveat of this system is that nicks introduced by the binding of a single Cas9-D10A/sgRNA complex to an off-target region could lead to off-target mutations [17,18,48,49]. To address this issue, the off-target mutation rates of Cas9-D10A transfected with one or two of its sgRNAs were measured and compared with the off-target mutation rate of wild-type Cas9 (WT-Cas9) at five potential off-target sequences. Deep sequencing revealed off-targeting (~3–35%) at all potential sites by WT-Cas9. However, off-targeting by Cas9-D10A transfected with one or two of its sgRNAs was not detected above background (Table 1). A second experiment measuring off-targeting for two different guide RNAs gave similar results. On-target mutation frequencies of up to 40% were observed in target sequences when the distance between the Cas9 nickase pairs were between −4 and 20 bp (Table 1).

In addition to the development of dual Cas9 platforms, other advances have been made to improve the specificity of CRISPR-Cas technology. It has recently been demonstrated that truncated sgRNAs (tru-gRNAs), which are deleted at the 5′ end resulting in a shorter 17–18 nucleotides sgRNA, are more sensitivity to mismatched bases. Thus, they display reduced off-target mutation rates while maintaining the efficiency of on-target modifications [17]. Conversely, the addition of two guanines at the 5′ end of sgRNAs greatly decreased off-target mutation rates; however, the GGX20 sgRNAs were less active at on-target sites [50]. An additional mechanism to decrease off-targeting is to reduce the amount of Cas9 and sgRNA DNA delivered to cells; however, this approach leads to a reduction in on-target cleavage [43,44].

A summary of the previously discussed genome-scale single and dual Cas systems can be found in (Table 1). All are excellent technologies with exciting implications for ongoing research. A clear trade-off for the impressively low off-target mutations rates of the dual Cas9 systems appears to be their lower on-target mutation rates relative to the single Cas9 systems. Depending on the needs of the investigator, the larger, but still small, degree of off-targeting by the single Cas9 systems may be an acceptable trade-off for their relatively higher on-target mutation rates. In addition to the global editing platforms discussed here, there are a plethora of other exciting CRISPR-Cas platforms for editing the genome on a smaller scale that have been developed and are evolving [9,50,51].

### CRISPR-Cas therapeutic potential

The ability to engineer genomic DNA in cells and organisms will lead not only to major advances in the investigation of disease processes but will also drive therapeutic innovations [52]. An exciting potential use of the CRISPR-Cas system is to correct disease-causing genetic mutations. For example, CRISPR-Cas9 genome editing has been used to correct mutations in a human cell line that causes cystic fibrosis and in murine zygotes that causes cataracts [25,26]. More recently, a rare, but fatal, genetic condition caused by a mutation of the fumarylacetoacetate hydrolase (FAH) gene in liver cells was corrected using CRISPR-Cas9 [27]. Components of the CRISPR-Cas9 system along with a homology-driven repair template were delivered to liver cells by hydrodynamic injection. The injection resulted in a subpopulation of liver cells (1/250) in which the wild-type sequence of the *Fah* gene had been knocked in replacing the mutated sequence. The repaired liver cells displayed expression of the wild-type Fah protein, exhibited a growth advantage over uncorrected cells, and expanded, resulting in a rescue of the body weight loss phenotype displayed by mutant mice.

CRISPR-Cas9-mediated genome editing was also used to correct a genetic mutation in mdx mice, the mouse model of Duchenne muscular dystrophy (DMD) [28]. Mdx mice (C57BL/10ScSn-Dmdmdx/J) contain a nonsense mutation in exon 23 of the mouse dystrophin gene introducing a premature stop codon, which leads to the absence of full-length dystrophin and a human DMD-like phenotype [53]. CRISPR-Cas9 plus a sgRNA targeting exon 23 of the dystrophin gene and a single-stranded oligodeoxynucleotide used as a template for HDR-mediated gene repair were injected into the pronucleus of mdx mouse zygotes. The injected zygotes were then implanted into pseudo-pregnant females and once born were monitored for defects in muscle structure and function. The pups were mosaic for the genetic correction (2 to 100%) likely reflecting that in some zygotes, the genetic correction occurred at some point after the one-cell stage. Interestingly, the degree of phenotypic rescue exceeded the degree of mosaicism. The authors point out that this likely reflects an advantage of the corrected cells and their contribution to regenerating muscle.

CRISPR-Cas9 technology has likewise shown promise as a therapeutic against viral infection. The clearance of intrahepatic hepatitis B viral (HBV) templates *in vivo* has been achieved using the CRISPR-Cas9 system [54]*.* This indicates the potential of CRISPR-Cas9 to be used as a therapeutic to eradicate persistent HBV infection in patients. Additionally, the CRISPR-Cas9 system was utilized to target specific sequences within the human immunodeficiency virus (HIV)-1 long terminal repeats promoter U3 region [55]. The targeted regions of the HIV genome were edited following introduction of Cas9/sgRNA expression. In microglial, promonocytic, and T lymphocytes latently infected with HIV, the CRISPR-Cas editing inactivated viral gene expression and replication. Importantly, off-target mutations were not detected in the host cells expressing Cas9/sgRNA and no genotoxicity was observed. A 9-kb fragment of integrated proviral DNA was completely excised from the host cell genome by Cas9/sgRNA editing. Additionally, continued expression of Cas9/sgRNA in host cells prevented HIV-1 infection of those cells. The authors note that this data suggests that Cas9/sgRNA can be engineered to provide a specific, effective, prophylactic, and therapeutic approach against HIV/AIDS.

Potential therapeutic advances in the field of oncology are also on the horizon. Chimeric antigen receptor (CAR) T cell therapy is a personalized cancer immunotherapy [56]. In CAR T therapy, a patient’s own T cells are collected, genetically engineered to produce receptors that recognize a specific protein (antigen) on tumor cells [57]. The T cells are then infused back into the patient where they use their engineered receptors to target and kill cancer cells that express the antigen on the surface of their membranes [56,57]. Novartis has recently gained rights to use Intellia’s CRISPR gene-editing technology to engineer chimeric antigen receptor T cells (CAR-Ts) for the purpose of developing CRISPR-Cas-based CAR therapies [58]. Novartis has also partnered with Caribou Biosciences “to research new CRISPR-based drug target screening and validation technologies” [58].

### More than just a nuclease

**Figure 3:**
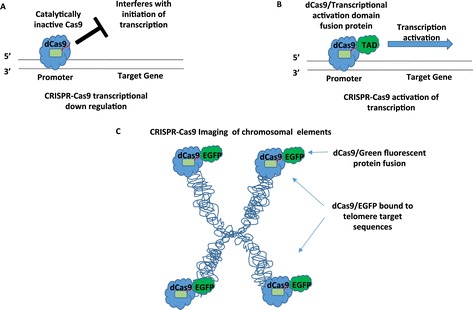
**Applications for the CRISPR-Cas9 system beyond gene editing. (A)** CRISPR-Cas9 as a tool for inhibiting transcriptional activation. sgRNAs can be used to direct the binding of catalytically inactive Cas9 (dCas9) to the promoter regions of genes. Once bound, dCas9 can interfere with transcriptional initiation of the gene and thus inhibit gene expression. **(B)** CRISPR-Cas9 to promote the transcription of a gene. sgRNAs can be used to direct the binding of a catalytically inactive Cas9 protein fused to a transcriptional activation domain (dCas9/TAD) to the promoter regions of genes. Once bound, dCas9/TAD can promote transcription of the target gene. **(C)** CRISPR-Cas9 to image various elements of the genome. sgRNAs can be used to direct the binding of catalytically inactive Cas9 fused to enhanced green fluorescent protein (dCas9/EGFP) to various elements of the genome. This technology can be used to image different elements of a chromosome, telomeres in this example, in live cells. Dynamic chromosomal changes during growth and replication can also be imaged.

Using the CRISPR-Cas system for genome editing is just the beginning of its utility. For example, CRISPR-Cas can be used to regulate the expression of genes. Catalytically inactive Cas9 (dCas9) guided to the promoter region of a gene can repress transcription by interfering with transcriptional elongation (Figure 3A) [59,60]. Transcriptional repression can be enhanced by fusing a transcriptional repression domain to dCas9 [61,62]. Likewise, dCas9 can be fused to a transactivation domain and be used to upregulate the expression of a gene (Figure 3B) [49,63-66].

Imaging of various elements in the genome of live cells can also be accomplished using the CRISPR-Cas9 system. An enhanced green fluorescent (EGFP)-tagged catalytically inactive Cas9 (dCas9) proteins can be guided to genomic elements via sequence-specific sgRNAs (Figure 3C) [67,68]. The dCas9-EGFP and modified sgRNAs designed to have increased stability and enhanced Cas9 binding were used to image telomere dynamics in living cells during elongation and disruption and cohesion of replicated loci on sister chromatids and their behaviors during mitosis. This technology allows for the visualization of chromatin dynamics in living cells with results that are on par with fluorescent in situ hybridization. The dCas9-EGFP imaging system has the potential to improve the capacity for studying the conformation and dynamics of native chromosomes in living cells. Beyond what has been highlighted here, there are many other applications of CRISPR-Cas beyond genome editing. The reader is referred to the following references that cover additional applications [50,51].

## Conclusions and implications for functional studies

The number of recent innovations to the CRISPR-Cas system has been enormous. CRISPR-Cas platforms continue to evolve with constantly improving sgRNA design, on-target specificity, and target sequence selection algorithms. With respect to cancer and other disease-related research, there is an urgent need for unbiased comprehensive functional studies of genes in disease. CRISPR-Cas9 is a scalable and effective new technology that can be used to knock out individual gene expression in large scale at the gene level and do so with minimal off-target effects. These types of large-scale unbiased loss-of-function studies will help to both separate genes that are drivers of disease from passengers and will create entirely new avenues for therapeutic investigation. Further, though many hurdles remain, CRISPR-Cas is beginning to show therapeutic potential for use in the correction of disease-causing genetic mutations. Finally, novel innovations in CRISPR-Cas technology beyond gene editing are expanding its utility as a tool for modulating gene expression and imaging chromatin dynamics in living cells. The continued innovation of programmable nuclease technologies such as CRISPR-Cas9 and their contributions to disease research and therapeutics will remain an exciting area of research to follow for the foreseeable future.
